# The effect of body mass index on quality of life in modified single stoma cutaneous ureterostomy or ileal conduit after radical cystectomy

**DOI:** 10.1002/cam4.6638

**Published:** 2023-10-30

**Authors:** Wan‐Jin Zhang, Xu‐Yun Huang, Bin Lin, Wen‐Cai Zheng, Zhi‐Bin Ke, Xiao‐Dan Lin, Jia‐Yin Chen, Hai Cai, Yun‐Zhi Lin, Ye‐Hui Chen, Qing‐Shui Zheng, Yong Wei, Xue‐Yi Xue, Xiao‐Dong Li, Ning Xu

**Affiliations:** ^1^ Department of Urology, Urology Research Institute, The First Affiliated Hospital Fujian Medical University Fuzhou China; ^2^ Department of Urology, National Region Medical centre, Binhai Campus of the First Affiliated Hospital Fujian Medical University Fuzhou China; ^3^ Fujian Key Laboratory of Precision Medicine for Cancer, the First Affiliated Hospital Fujian Medical University Fuzhou China

**Keywords:** bladder cancer, body mass index, ileal conduit, modified single stoma cutaneous ureterostomy, quality of life

## Abstract

**Objective:**

To explore the influence of postoperative body mass index (BMI) change on postoperative quality of life (QOL) in patients undergoing radical cystectomy (RC) plus modified single stoma cutaneous ureterostomy (MSSCU) or ileal conduit (IC).

**Methods:**

Patients were divided into two groups according to different BMI change patterns: patients experiencing an elevated postoperative BMI level, along with a clinically significant increase in their BMI (an increase of more than 10%) were categorized as Group 1, while patients experiencing a decrease postoperative BMI level, along with a clinically significant reduction in their BMI (a decrease of more than 5%) were categorized as Group 2. Spearman correlation analysis was used to examine the correlations between quality‐of‐life scores and postoperative clinical parameters.

**Results:**

Spearman correlation analysis showed that postoperative BMI, late complications and catheter‐free state were significantly associated with postoperative global QoL and symptom scale in MSSCU and postoperative global QoL and physical scale in IC patients. Additionally, postoperative BMI, catheter‐free state and the use of adjuvant therapy were associated with bad performance in many scales of QoL like body image, future perspective, social scale, future perspective (MSSCU), and abdominal bloating (IC) (Table 2, *p*<0.05). Patients in Group 2 with significant weight loss had a better Global QoL, a lower rate of stomal stricture and a higher catheter‐free state compared with those in Group 1 in both IC and MSSCU patients. MSSCU patients in Group 2 could achieve a comparable Global QoL as to IC patients in Group 1.

**Conclusion:**

Controlling the substantial increase in body weight after surgery contributes to improving QoL, reducing the occurrence of stomal stricture, and ensuring a postoperative catheter‐free state in BCa patients undergoing MSSCU.

## 1 INTRODUCTION

Bladder cancer (BCa) is considered as the second common malignancy of the genitourinary system.^1^ Radical cystectomy (RC) plus urinary diversion (UD) remains the gold standard surgical treatment for muscle invasive bladder cancer (MIBC) and high‐risk non‐ muscle invasive bladder cancer (NMIBC).^2^ Recently, the orthotopic UD (such as orthotopic neobladder) has been used more frequently from the perspective of postoperative quality of life (QoL).^3^, ^4^ However, this procedure is technically challenging for surgeons and has a higher incidence of complication.^5^ Besides, it was also contradictorily reported that the long‐term health‐related QoL of orthotopic neobladder, especially those with postoperative urinary incontinence, was not superior to that of ileal conduit (IC), and cutaneous ureterostomy (CU).^6^, ^7^ Patients receiving incontinent UD (including IC and CU) have shorter operative time, less blood loss, lower transfusion rates, lower need for intensive care compared with those receiving orthotopic UD.^8^ The QoL of patients receiving incontinent UD was significantly affected by the presence of stoma and stoma‐related complications.^9^ There is still a debate on which type of incontinent UD could provide better QoL for patients undergoing RC.^10^ For younger patients, the postoperative QoL of patients receiving IC had a marginal benefit compared with those receiving CU.^11^ However, recent findings revealed that the QoL and postoperative complications of patients receiving unilateral CU was superior to those receiving IC.^12^, ^13^

It has been demonstrated that body mass index (BMI) shifts after treatment have a significant impact on the QoL.^14^, ^15^ However, there was no study reporting the impact of BMI shifts on QoL after RC plus IC or CU. In this study, we collected the questionnaire survey data of the patients who underwent RC plus modified single stoma cutaneous ureterostomy (MSSCU) or IC to explore the influence of postoperative BMI on postoperative QoL, which might provide valuable references on carrying out early effective intervention after RC and preventing the decrease of postoperative QoL.

## 2 PATIENTS AND METHODS

### 2.1 Patients

We retrospectively analyzed a total of 160 BCa patients receiving laparoscopic RC plus extracorporeal UD in the First Affiliated Hospital of Fujian Medical University from January 2017 to February 2021. All patients underwent laparoscopic RC plus MSSCU or IC.^16^, ^17^ In patients included in this study, MSSCU was recommended for elderly patients, those in poor health conditions, or those with contraindications to ileal manipulation; otherwise, IC would be recommended. All patients were followed up 1 year after surgery.

BMI was calculated as follows: weight/height ^2^ (kg/m^2^). The degree of obesity is divided into four classes based on BMI: underweight (<18.50 kg/m^2^), normal (18.50–22.99 kg/m^2^), overweight (23.00–24.99 kg/m^2^), and obese (>25.00 kg/m^2^). Patients were divided into two groups according to the change in BMI level and the percentage change of BMI: Patients in Group 1 had an elevated BMI level and an increase of more than 10% and patients in Group 2 had a decreased BMI level and a reduction of more than 5%. In further analysis, we would exclude patients with no significant change in BMI.

Inclusion criteria^18^: ①18–80 years old; normal ureteral structure and function as indicated by preoperative imaging; normal renal function as indicated by preoperative laboratory examinations; ② postoperative pathological report indicating the diagnosis of confined BCa without distant metastasis; ③0–1 points of ECOG score within 1 year after surgery; ④adequate educational level of to understand and complete the questionnaires correctly. Exclusion criteria: ①a history of neoadjuvant chemotherapy; ②lymph node metastasis indicated by pathology; ③combining with other types of tumors; ④tumor recurrence or metastasis or deaths from any cause within 1 year after surgery; ⑤< 6 months of follow‐up duration from operation (disease‐related factors or early postoperative mental state would affect the assessment of QoL)^19^; ⑥histories of hypertension, heart disease, diabetes, cerebrovascular accidents, psychiatric illness or/and other long‐term chronic diseases.

### 2.2 Urinary diversion technique

#### 2.2.1 MSSCU

After bladder resection, the surgeon closed the laparoscopic surgical system, then removed the trocar and pulled out the ureter from the incision to prepare for the MSSCU: single J stent was inserted into the dissociative proximal ureter and fixed properly with thread. A “S” shaped incision for ureterostomy was made at the junction of the middle third and the outer third of the line between the right anterior superior iliac spine and umbilicus. The aponeurosis of the external abdominal oblique and internal abdominal oblique was disintegrated, and hemostasis was well ensued. The bilateral ureters were pulled out of the abdominal wall through this incision and exposed outside for 3 cm. The two ureters were opened 2 cm longitudinal. Then, absorbable 4–0 Ethicon thread was used for side‐to‐side anastomosis for the sake of forming a common opening. Then the reconstructed single‐stoma ureter was returned into the abdominal cavity. The surgeon restarted the laparoscopic system and finished the rest of the work. The remaining colostomy procedure was completed in vitro: the ureteral plasmomuscular layer, aponeurosis of the external oblique and skin incision were everted and fixed with 3–0 silk thread.

#### 2.2.2 IC

In our center, the Wallace surgical technique was performed on all patients. After bladder resection, surgeon closed the laparoscopic surgical system, then removed the trocar and pulled out the ureter and ileal loop used to make catheter from the incision to prepare for the IC: a 15–20 cm intestinal loop equipped with mesentery was intercepted from the terminal ileum about 15 cm from the ileocecal junction which has been previously marked in the laparoscopic setting. Then, intestinal continuity was restored. The free intestinal loop was rinsed with physiological saline and iodophor repeatedly. End‐to‐side ileal anastomosis was performed on the ureters and the proximal ends were closed well. Then the prepared IC was returned into the abdominal cavity. The surgeon restarted the operating system and finished the rest of the work. The remaining colostomy procedure was completed in vitro: Stoma was made at one‐third of the outside junction between the right anterior superior iliac spine and the umbilicus. The aponeurosis of the external abdominal oblique and the internal abdominal oblique were treated like the previous processing. The intestinal loop was pulled out through the stoma, about 5 cm above the skin, and formed an oval‐shaped papillary stoma.

After 3 months of postoperative urinary tract patency, the ureteral stent was removed.

### 2.3 QoL assessment and follow‐up methods

All patients provided written informed consent. The study was approved by the Ethics Committee of the First Affiliated Hospital of Fujian Medical University and was conducted in accordance with the principles of research involving human subjects as stated in the Helsinki Declaration and Good Clinical Practice Guidelines.

Questionnaire: QoL was assessed using the European Organization for Research and Treatment of Cancer QoL Questionnaire Core30 (EORTC QLQ‐C30) and the BCa specific instruments (EORTC QLQ‐BLM30).^19^, ^20^, ^21^ There are two questions on a scale of one to seven score while the other questions are recorded on a scale of one to four score (1 = not at all; 2 = a little; 3 = quite a bit; 4 = very much). As recommended by the EORTC scoring manual, we linearly converted all variables to 0–100 points. The scoring principles of the questionnaire are as follows: (1) the original score is composed of the average scores of each item; (2) the original score is standardized by linear transformation, making the score range from 0 to 100. For functional items, the higher the score, the higher the functional level. For symptoms/individual items, a higher score means a higher level of symptom/problem.

Searching the patient database and consulting the patient's medical record, consent from each patient was obtained after introducing the purpose and methods of this study. The distribution and collection of questionnaire: ① mail questionnaires with replying postage to patients who agreed to participate in this study; ② send questionnaires by E‐mail to those qualified to reply by E‐mail; ③ telephone inquiry for those without valid address; ④ outpatient follow‐up. At the end of the follow‐up period, any complications observed between 3 months and 1 year after surgery were recorded as late complications.

### 2.4 Statistical analysis

Statistical analysis was performed by SPSS 22.0 software (IBM Corp., Armonk, NY, USA). The quantitative data were analyzed by *t* test or Wilcoxon Two‐Sample test which were presented as mean ± standard deviation or median (IQR). The categorical data were analyzed by chi‐square test or Fisher's exact method which were presented as number (percentage). Spearman correlation analysis was used to explore the correlation between postoperative clinical parameters and QoL. *p* < 0.05 was considered statistically significant.

### 2.5 Patient and public involvement

Patients or the public were not involved in the design, or conduct, or reporting, or dissemination plans of our research. The study protocol was approved by the institutional review board of the Ethics Committee of the First Affiliated Hospital of Fujian Medical University (IRB No. MTCA, ECFAH of FMU [2015]084–1).

## 3 RESULTS

Among 160 patients, six patients were lost to follow‐up, eight patients died of postoperative tumor recurrence or metastasis, and five patients refused to participate. Finally, a total of 141 patients successfully completed the evaluation of QoL. 121(85.8%) cases were male and 20(14.2%) cases were female. The mean age was 63.8 years. Among them, 65 patients underwent MSSCU with a mean age of 67.71 years and 76 patients underwent IC with a mean age of 60.52 years. There were 125 cases of transitional cell carcinoma, 12 cases of adenocarcinoma, and 5 cases of transitional cell carcinoma with adenocarcinoma. Nearly (55/141) 40% of patients were overweight or obese before the surgery, and following the surgery, this proportion increased to 44% (62/141). The comparison of clinical data between MSSCU group and IC group was presented in Table 1. The median age of patients who underwent MSSCU was higher than that of those who underwent IC. There were significant differences in stomal stricture and catheter‐free state between MSSCU group and IC group. MSSCU group has a higher stomal stricture rate (29.8% vs. 11.2%, *p*<0.05) and lower catheter‐free state (72.3% vs. 89.5%, *p*<0.05) (Table1). The results of Spearman correlation analysis showed that postoperative BMI, late complications, catheter‐free state were correlated with postoperative global QoL and symptom scale in MSSCU patients and postoperative global QoL and physical scale in IC patients. Additionally, postoperative BMI was correlated with lower body image score in both groups. Patients with catheters were associated with lower scores in social functioning, sexual function, future perspective (for those underwent MSSCU), and body image (for those underwent IC). The use of adjuvant therapy decreased global QoL in MSSCU patients. (Table 2, *p*<0.05). In the further analysis, the 92 patients were categorized into Group1 (*n* = 42) and Group 2 (*n* = 50) according to different patterns of BMI change. In Group 1, global QoL, physical functioning scores and future perspective scores of MSSCU patients (*n* = 22) were lower than that of IC patients (*n* = 20) while symptoms functioning scores were significantly increased than that of IC patients. In Group 2, the social functioning score of MSSCU patients (*n* = 19) was increased compared with that of IC patients (*n* = 31) (Table3, *p*<0.05). The incidence of stomal stricture and catheter‐free state was statistically different between the patients in the MSSCU and IC groups within both Group 1 and Group 2. Specifically, patients in the IC group had a higher rate of achieving a catheter‐free state and a lower incidence of stomal stricture. Among patients in Group 2 with significant weight loss, patients had lower rate of stomal stricture (MSSCU: 36.8% vs. 45.5%, IC: 15% vs. 9.7%, Table 4) and higher rate of achieving catheter‐free state (MSSCU: 63.2% vs. 50.0%, IC: 90.3% vs. 85.0%, Table 4).

**TABLE 1:** Clinical characteristics of bladder cancer patients classified by the type of urinary diversion.

	MSSCU (*N* = 65)	IC (*N* = 76)	*p*
Age (mean, IQR)	70.00(13.80)	60.00 (13.75)	<0.001
Gender (*n*, %)			0.708
Male	55 (84.6)	66 (86.8)	
Female	10 (15.4)	10 (13.2)	
Preoperative BMI, kg/m^2^(*n*, %)			0.062
Underweight (<18.50)	9 (13.8)	3 (3.9)	
Normal (18.50–22.99)	35 (53.8)	39 (51.3)	
Overweight (23.00–24.99)	12 (18.5)	21 (27.6)	
Obese (>25.00)	9 (13.9)	13 (17.2)	
Preoperative hydronephrosis (*n*, %)	8 (12.3)	10 (13.1)	0.708
Performance status (ECOG, *n*, %)			0.979
0	54 (83.1)	60 (78.9)	
1	11 (16.9)	16 (21.1)	
Hypertension (*n*, %)	5 (7.7)	8 (10.5)	1.000
Heart disease (*n*, %)	4 (6.2)	7 (9.2)	0.708
Diabetes (*n*, %)	5 (7.7)	11 (14.5)	0.529
Stage (pTNM—UICC) (*n*, %)			0.169
0‐I	19 (29.2)	33 (43.4)	
II	18 (27.7)	23 (30.3)	
III–IV	25 (38.5)	20 (26.3)	
Grade (*n*, %)			0.168
G1–G2	13 (20.0)	14 (18.4)	
G3–G4	32 (49.2)	3 (39.5)	
Unknown	20 (30.8)	32 (42.1)	
Neoadjuvant chemotherapy/radiotherapy (*n*, %)			0.164
No	55 (84.6)	70 (92.1)	
Yes	10 (15.4)	6 (7.9)	
Postoperative BMI, kg/m^2^ (*n*, %)			0.551
Underweight (<18.50)	13 (20.0)	19 (25.0)	
Normal (18.50–22.99)	28 (43.1)	19 (25.0)	
Overweight (23.00–24.99)	13 (20.0)	24 (31.6)	
Obese (>25.00)	11 (16.9)	14 (18.4)	
Postoperative performance status (ECOG) (*n*, %)			0.602
0	55 (84.6)	66 (86.8)	
1	10 (15.4)	10 (13.2)	
Adjuvant therapy (*n*, %)			0.708
No	55 (84.6)	66 (86.8)	
Yes	10 (15.4)	10 (13.2)	
Late complications (*n*, %)			
Acute pyelonephritis (*n*, %)	8 (12.3)	10 (13.1)	0.881
Postoperative hydronephrosis (*n*, %)	10 (15.4)	17 (22.4)	0.297
Stone formation (*n*, %)	12 (18.5)	8 (10.5)	0.906
Stomal stricture (*n*, %)	19 (29.2)	9 (11.8)	0.010*
Catheter‐free state (*n*, %)	47 (72.3)	68 (89.5)	0.009*

Abbreviations: BMI, body mass index; IC, Ileal conduit; MSSCU, modified single stoma cutaneous ureterostomy.

* *p*<0.05.

**TABLE 2:** Spearmen correlation analysis for postoperative status.

	Postoperative BMI^a^		ECOG^b^		Late complications^c^		Catheter‐free state^d^		Adjuvant therapy^e^	
	*γ*	*p*‐ Value	*γ*	*p*‐ Value	*γ*	*p*‐ Value	*γ*	*p*‐ Value	*γ*	*p*‐ Value
MSSCU										
EORTC QLQ‐C30										
Global quality of life	−0.285	0.021*	−0.161	0.180	−0.251	0.044*	−0.257	0.039*	−0.245	0.049*
Physical scale	0.012	0.923	−0.122	0.332	−0.005	0.967	−0.250	0.044*	−0.181	0.150
Role scale	0.050	0.695	0.056	0.657	0.038	0.766	0.023	0.856	0.095	0.449
Emotional scale	−0.049	0.696	−0.222	0.075	−0.107	0.398	−0.186	0.138	−0.073	0.565
Cognitive scale	−0.137	0.278	0.110	0.382	0.143	0.256	−0.239	0.055	0.157	0.212
Social scale	−0.193	0.124	−0.119	0.347	−0.005	0.968	−0.326	0.008*	−0.063	0.621
Symptoms scale	0.245	0.049*	0.171	0.174	0.356	0.004*	0.332	0.007*	−0.193	0.123
EORTC QLQ‐BLM30										
Future perspective	−0.194	0.121	−0.095	0.452	−0.095	0.452	−0.363	0.003*	−0.170	0.175
Abdominal bloating/flatulence	0.132	0.294	0.145	0.249	0.102	0.420	0.120	0.449	0.406	0.001
Body image	−0.399	0.001*	−0.096	0.448	−0.094	0.455	−0.202	0.107	−0.364	0.003*
Sexual functioning	−0.213	0.089	−0.043	0.731	−0.106	0.401	−0.371	0.002*	−0.070	0.577
IC										
EORTC QLQ‐C30										
Global quality of life	−0.240	0.037*	0.044	0.703	−0.282	0.002*	−0.410	<0.001*	0.044	0.703
Physical scale	−0.259	0.024*	−0.063	0.587	−0.265	0.039*	−0.431	<0.001*	−0.063	0.587
Role scale	0.147	0.207	−0.010	0.930	0.079	0.565	0.003	0.981	−0.010	0.930
Emotional scale	0.101	0.386	0.146	0.207	0.080	0.562	0.108	0.354	0.146	0.207
Cognitive scale	−0.213	0.064	0.050	0.667	0.044	0.698	0.119	0.304	0.050	0.667
Social scale	−0.012	0.916	0.197	0.088	0.208	0.046*	0.056	0.632	0.197	0.088
Symptoms scale	0.282	0.014*	0.070	0.547	0.329	0.004*	0.024	0.840	0.070	0.547
EORTC QLQ‐BLM30										
Future perspective	−0.050	0.668	−0.072	0.537	0.041	0.722	−0.064	0.583	−0.072	0.537
Abdominal bloating/flatulence	0.055	0.636	−0.068	0.560	0.182	0.069	0.366	0.001*	−0.068	0.560
Body image	−0.233	0.043*	−0.088	0.449	−0.071	0.540	−0.284	0.013*	−0.088	0.449
Sexual functioning	−0.228	0.048*	0.113	0.329	−0.137	0.236	−0.354	0.002*	0.113	0.329

*Note*: Group 1: patients with an elevation in their postoperative BMI class and a 10% increase in their postoperative BMI.

*Note*: Group 2: patients with a decrease in their postoperative BMI class and a 5% reduction in their postoperative BMI.

Abbreviations: MSSCU, modified single stoma cutaneous ureterostomy; IC, Ileal conduit.

* *p*<0.05.

^a^ 1 = (<18.50 kg/m^2^), 2 = (18.50 ~ 22.99 kg/m^2^), 3 = (23.00 ~ 24.99 kg/m^2^), 4 = (>25.00 kg/m^2^).

^b^ 1 = 0, 2 = 1.

^c^ 1 = one complication, 2 = two complications, 3 = three complications, 4 = four complications, 5 = five complications.

^d^ 0 = Catheter‐free, 1 = Catheter‐ placement.

^e^ 0 = no, 1 = yes.

**TABLE 3:** Comparison of post‐operative QoL according to BMI shifting in bladder cancer patients.

	Group 1 (*n* = 42)		*p*	Group 2 (*n* = 50)		*p*
	MSSCU (*n* = 22) Median (IQR)	IC (*n* = 20) Median (IQR)		MSSCU (*n* = 19) Median (IQR)	IC (*n* = 31) Median (IQR)	
EORTC QLQ‐C30						
Global quality of life	49.9 (13.3)	52.7 (24.8)	0.045*	52.6 (10.3)	59.1(15.2)	0.857
Physical functioning	52.6 (10.3)	59.1 (15.2)	0.027*	70.1 (8.1)	73.8 (6.3)	0.150
Role functioning	72.2 (13.6)	73.1 (7.3)	0.232	50.4(22.1)	53.1 (14.4)	0.503
Emotional functioning	70.2 (12.2)	69.3 (10.5)	0.203	71.6 (10.5)	73.0 (7.2)	0.066
Cognitive functioning	74.4 (11.0)	75.7 (5.1)	0.753	62.8 (32.2)	59.3 (28.9)	0.110
Social functioning	63.5 (20.1)	54.5 (19)	0.141	57.6 (15.8)	52.5 (15.6)	0.049*
Symptoms functioning	54.5 (33.3)	50.1 (20.0)	0.017*	73.8 (9.8)	67.8 (18)	0.190
EORTC QLQ‐BLM30						
Future perspective	45.9 (41.1)	56.4 (38.3)	0.035*	52.1 (27.3)	38.5 (27.4)	0.259
Abdominal bloating/flatulence	31.5 (36.1)	30.8 (20.5)	0.096	53.7 (52.2)	34.5 (36.9)	0.289
Body image	38.6 (34.5)	35.0 (36.3)	0.980	28.5 (34.7)	28.1 (14.7)	0.187
Sexual functioning	27.5 (24.8)	25.5 (39.5)	0.351	27.5 (33.7)	25.9 (17.8)	0.757

* Wilcoxon two‐sample test, *p*<0.05.

Note: Group 1: patients with an elevation in their postoperative BMI class and a 10% increase in their postoperative BMI.

Note: Group 2: patients with a decrease in their postoperative BMI class and a 5% reduction in their postoperative BMI.

**TABLE 4:** Long‐term postoperative conditions of the two groups of patients according to the BMI shifting.

	Group 1 (*n* = 42)		*p*	Group 2 (*n* = 50)		*p*
	MSSCU (*n* = 22)	IC (n = 20)		MSSCU (*n* = 19)	IC (*n* = 31)	
Late complications (*n*, %)						
Acute pyelonephritis	1 (4.5)	4 (20.0)	0.286	4 (21.1)	3 (9.7)	1.000
Postoperative hydronephrosis	3 (13.7)	5 (25.0)	0.587	1 (5.3)	6 (19.4)	0.330
Stone formation	4 (18.2)	4 (20.0)	1.000	2 (10.5)	6 (19.4)	0.668
Stomal stricture	10 (45.5)	3 (15.0)	0.033*	7 (36.8)	3 (9.7)	0.049*
Catheter‐free state (*n*,%)	11 (50.0)	17 (85.0)	0.016*	12 (63.2)	28 (90.3)	0.049*

* Chi square test, *p*<0.05.

*Note*: Group 1: patients with an elevation in their postoperative BMI class and a 10% increase in their postoperative BMI.

*Note*: Group 2: patients with a decrease in their postoperative BMI class and a 5% reduction in their postoperative BMI.

## 4 DISCUSSION

In BCa patients, the postoperative prognosis depends on multiple dimensions. In the population of cancer patients, most tumors seem to be closely related to obesity. It is known that obesity increases the risk of chronic disease and malignancy, while also being linked to patients' frailty.^22^, ^23^, ^24^, ^25^ Obesity also increases postoperative complications rate and mortality, resulting in declining QoL.^26^ The adoption of different surgical techniques also plays a crucial role in influencing survival benefits and complication. Additionally, the long‐term complication, especially for patients wearing urinary bags, may have a significant impact on patients' sense of dignity and self‐perception. The QoL questionnaire was reported as a useful tool to evaluate the physical and psychological status of patients after surgery.^27^ In recent years, increasing attention is being paid to postoperative QoL, with the significant prolongation of lifetime, the progress of comprehensive treatment of cancer and the continuous improvement of QoL assessment tools.^11^, ^28^ Compared with the symptom scale or QLQ‐BLM30 scale, the functional scale may be more influenced by the psychological domain than the somatic domain.^15^ The assessment of QoL plays an increasingly important role in the prognosis evaluation of various malignancies.^28^, ^29^ Through a comprehensive QoL questionnaire assessment, our study aims to explore the impact of postoperative weight changes on the QoL of BCa patients and discusses the correlation between different surgical techniques, long‐term complications and QoL.

According to baseline characteristics of the patients, it was evident that the average age of BCa patients was more than 60 years old, as the median age of MSSCU patients was70 years old, with nearly 40% of the patients were overweight or obese, highlighting the concerning prevalence of obesity among elderly cancer patients.^30^ For this kind of elderly and obese patients, the decreased skeletal muscle content and frailty significantly diminished their capacity to tolerate RC and urinary diversion surgery and impacted perioperative outcomes and long‐term postoperative prognosis. Related studies had shown that frail patients have an increased rate of perioperative complications and an increased risk of death after surgery.^31^, ^32^ While effective weight management and lifestyle intervention can reduce the complications caused by obesity, improving the frailty of patients and their QoL.^33^ In our study, we conducted an analysis of quality‐of‐life scores for patients experiencing postoperative weight gain and confirmed the positive effect of weight loss on postoperative QoL. However, we did not perform a specific analysis for patients with frailty at this time. To provide a more comprehensive comparison of the quality‐of‐life benefits of surgery for frail patients, further research was deemed necessary.

Spearman's correlation analysis found that global QoL, physical scale, and symptoms scale were significantly associated with postoperative BMI, late complications, and catheter‐ free state. Postoperative BMI was negatively correlated with body image in two groups. The patient with catheter also performed poorly in social scale, future perspective, body image, and sexual function. We also revealed that those patients with clinically significant decrease BMI had a better QOL in both groups (MSSCU: 52.6 vs. 49.9; IC: 59.1 vs. 52.7 Table3). Hence, Patients receiving MSSCU would have the opportunity to achieve a comparable QoL compared with those receiving IC under the condition of weight management and lifestyle intervention. Previous studies have demonstrated that the promotion of perioperative exercise and the management of BMI played an important role in postoperative QoL. The loss of weight and the increase of exercise even have a positive impact on the overall survival of cancer patients.^14^, ^28^, ^34^ Our findings also suggested that lifestyle interventions are of great importance to improve the QoL in patients undergoing RC plus UD.

In addition, our study also revealed that the patients with postoperative decrease in BMI have a lowered rate of stomal stricture and improved the rate of catheter‐free state in MSSCU patients, which is one of the vital causes that the QoL of MSSCU. In patients requiring RC plus UD, CU was considered as the simplest and safest method of urinary diversion. CU could significantly reduce the incidence of postoperative gastrointestinal complications and decrease operation time in comparison with IC. Therefore, CU is especially applicable to elderly patients and those with systemic comorbidities.^13^, ^35^ However, the high incidence of postoperative stomal stricture limits the application of CU.^36^, ^37^ The ischemia of distal ureter is regarded as one of the main factors leading to stomal stricture. Stomal stricture and long‐term indwelling of ureteral stents would significantly decrease the QoL of BCa patients.^38^ There were various modified CU technique contributing to decreasing the incidence of postoperative stomal stricture, such as reducing abdominal tunnel length, preserving the fascia between spermatic cord and ureter, and modified stoma design.^39^, ^40^, ^41^ Arman^16^ revealed that MSSCU could not only reduce the incidence of stomal stricture but gain similar QoL compared with IC. Consistent with previous studies, we also performed MSSCU for patients requiring CU in order to minimize the incidence of postoperative stenosis.^42^

Careful dissection and retention of the peritoneum connected to the ureter, adequate length of abdominal wall tunnel, prevention of ureter knotting, prevention of ureter squeezing when crossing the abdominal wall, formation of papillostomy and delayed removal of stent tubes (6–12 weeks) were the vital factors for tubeless status after CU.^39^, ^40^, ^41^, ^42^ Previous studies also demonstrated that modified CU could achieve a significantly higher rate (76.9%) of tubeless status.^36^, ^42^, ^43^ We also utilized the improved procedures of previous studies to improve the success rate of postoperative tubeless status. Consistent with previous studies,^42^ we found that MSSCU is a vital guarantee of high success rate of postoperative tubeless status.

In this study, obesity was found to be a key factor for stomal stricture and the reduction of tubeless rate in MSSCU patients. Indeed, at the early postoperative stage, there may be a short‐term weight loss in patients. However, when BMI recovers or even reaches the level of overweight, abdominal subcutaneous fat and visceral fat would increase, which would lead to the pulling and tension of the ureter, the compression of the lumen and the extrusion of the ureteral skin stoma. These factors would increase the incidence of ureteral stricture and the probability of postoperative tubeless failure.^36^, ^43^ Therefore, patients undergoing MSSCU should receive more rigorous weight management compared with healthy individuals and patients undergoing IC. Patients undergoing MSSCU should be fully informed that their QoL would be maintained or improved under the condition of normal or decreased BMI after surgery.

Adjuvant chemotherapy might affect postoperative QoL. However, the course of adjuvant chemotherapy had ended 1 year after surgery; hence, adjuvant chemotherapy may not have a significant effect on QoL.^15^ Currently, there were few studies focusing on the effect of adjuvant chemotherapy after RC on the QoL. Our study revealed that adjuvant chemotherapy did not exist a significant effect on postoperative QoL 1 year after RC on patient underwent MSSCU.

RC (including open RC (ORC), laparoscopic RC (LRC), robotic assisted RC (RARC)) plus UD represents the “golden standard” treatments in patients with MIBC^4^, ^44^ There have been several studies comparing the patients QoL among different surgical technique. A single‐center three‐arm trial revealed that the QoL was comparable among ORC, LRC and RARC, but LRC has lower the 30‐day complication rate comparing with ORC and RARC (ORC: 70%; RARC: 55%; LRC: 26%).^45^ Jue et al. summarized that the QoL outcomes were similar between RARC and ORC.^46^ Fuschi et al. revealed that the rates of complications of intracorporeal UD were 37.4% of single stoma ureterocutaneostomy and 57.6% of IC.^47^ In our study, all patients underwent laparoscopic RC plus extracorporeal UD and the rate of late complication was 42.9% (MSSCU) and 43.5% (IC), which was nearly consistent with previous study.

There are several shortcomings in this study. Firstly, this is a retrospective study at single center and the primary limitation is the relatively small sample size. Additionally, data related to preoperative body circumference and frailty assessment were not consistently collected for hospitalized patients at our center. However, future research with a more comprehensive study design and a larger sample size is warranted. Secondly, the number of patients receiving orthotopic neobladder was small in our centre and could not meet the requirement of statistical analysis. The impact of BMI on QoL in patients receiving orthotopic urinary diversion could not be investigated. Thirdly, assessment of QoL using questionnaire survey existed potential bias. Patients might try to attract doctors' attention to their illness by giving inaccurate answers or obsequious answers.^19^, ^48^ Fourthly, there were various factors affecting QoL, including preoperative patient characteristics, surgical factors, pathological features, and psychological state, etc. The change of BMI is merely one of the negative factors affecting the QoL after surgery.^28^, ^49^ When evaluating postoperative QoL, the confusing of health and physical ability with QoL should be avoided.^29^, ^38^ In our study, to reduce bias, only patients with an ECOG score of 0–1 were included. Finally, compared with Western populations, the BMI of Asian populations was significantly decreased.^15^ Our results should be verified in Western populations.

## 5 CONCLUSION

Maintaining lower postoperative BMI contributes to improving QoL, reducing the occurrence of stomal stricture, and ensuring postoperative catheter‐free state in BCa patients undergoing MSSCU. The management of calorie intake, the maintenance of physical exercise would prevent MSSCU patients from having a worse QoL compared with IC patients after surgery. Surgeons should explain to patients that both MSSCU and IC would not decrease postoperative QoL.

## AUTHOR CONTRIBUTIONS

**Wan‐Jin Zhang:** Writing – original draft (equal). **Xu‐Yun Huang:** Writing – original draft (equal). **Bin Lin:** Writing – original draft (equal). **Wen‐Cai Zheng:** Writing – original draft (equal). **Zhi‐Bin Ke:** Formal analysis (equal). **Xiao‐Dan Lin:** Data curation (equal). **Jia‐Yin Chen:** Formal analysis (equal). **Hai Cai:** Formal analysis (equal). **Yun‐Zhi Lin:** Data curation (equal). **YeHui Chen:** Data curation (equal). **QingShui Zheng:** Conceptualization (equal); methodology (equal). **Yong Wei:** Methodology (equal); visualization (equal). **XueYi Xue:** Methodology (equal). **Xiao‐Dong Li:** Project administration (equal); writing – review and editing (equal). **Ning Xu:** Project administration (equal); writing – review and editing (equal).

## FUNDING INFORMATION

This study was supported by the Fujian Province Finance Project (No. 2020B018), Natural Science Foundation of Fujian Province (No.2021J01715), the “Eyas Plan” Youth Top‐notch Talent Project of Fujian Province (No. SCYJHBJRC‐XN2021), and Class B Talent Research Project of the First Affiliated Hospital of Fujian Medical University (No. YCRC‐B‐XN2022).

## CONFLICT OF INTEREST STATEMENT

All authors declare no conflict of interest.

## ETHICS STATEMENT

This study was approved by the Ethics Committee of the First Affiliated Hospital of Fujian Medical University and written informed consent was provided.

## Data Availability

The datasets used and/or analyzed during the current study are available from the corresponding author on reasonable request.
